# Genome-Wide Characterization and Expression Analysis of *bZIP* Gene Family Under Abiotic Stress in *Glycyrrhiza uralensis*

**DOI:** 10.3389/fgene.2021.754237

**Published:** 2021-10-05

**Authors:** Yuxuan Han, Zhuoni Hou, Qiuling He, Xuemin Zhang, Kaijing Yan, Ruilian Han, Zongsuo Liang

**Affiliations:** ^1^ The Key Laboratory of Plant Secondary Metabolism and Regulation of Zhejiang Province, College of Life Sciences and Medicine, Zhejiang Sci-Tech University, Hangzhou, China; ^2^ Tasly R&D Institute, Tasly Holding Group Co., Ltd., Tianjin, China; ^3^ Institute of Landscape and Plant Ecology, The School of Engineering and Architecture, Zhejiang Sci-tech University, Hangzhou, China

**Keywords:** bZIP transcription factor, licorice, abiotic stress, interaction network, expression

## Abstract

*bZIP* gene family is one of the largest transcription factor families. It plays an important role in plant growth, metabolic, and environmental response. However, complete genome-wide investigation of *bZIP* gene family in *Glycyrrhiza uralensis* remains unexplained. In this study, 66 putative *bZIP* genes in the genome of *G. uralensis* were identified. And their evolutionary classification, physicochemical properties, conserved domain, functional differentiation, and the expression level under different stress conditions were further analyzed. All the members were clustered into 13 subfamilies (A–K, M, and S). A total of 10 conserved motifs were found in GubZIP proteins. Members from the same subfamily shared highly similar gene structures and conserved domains. Tandem duplication events acted as a major driving force for the evolution of *bZIP* gene family in *G. uralensis*. Cis-acting elements and protein–protein interaction networks showed that *GubZIPs* in one subfamily are involved in multiple functions, while some *GubZIPs* from different subfamilies may share the same functional category. The miRNA network targeting GubZIPs showed that the regulation at the transcriptional level may affect protein–protein interaction networks. We suspected that domain-mediated interactions may categorize a protein family into subfamilies in G. uralensis. Furthermore, the tissue-specific gene expression patterns of GubZIPs were analyzed using the public RNA-seq data. Moreover, gene expression level of 66 bZIP family members under abiotic stress treatments was quantified by using qRT-PCR. The results of this study may serve as potential candidates for functional characterization in the future.

## Introduction

Basic leucine zipper (bZIP) is a transcription factors (TFs) family and widely distributed in eukaryotes. The structure of bZIP protein is defined by the conserved bZIP protein and often acts as a dimer (Mair et al., 2015). They are abundant in different species. A total of 78 *bZIP* genes have been found in *Arabidopsis* (*Arabidopsis thaliana*) (Dröge-Laser et al., 2018), 65 in potato (*Solanum tuberosum* L.) (Zhao et al., 2020), 132 in tobacco (*Nicotiana tabacum* L.) (Li et al., 2021), 57 in *Medicago sativa* (Liu et al., 2021), and 86 in rice (E et al., 2014). The *bZIP* TF has a specific sequence consisting of a fixed motif N-X_7_-R/K and a leucine zipper that binds to the alkaline region. In the leucine zipper region, the highly conserved heptad repeats of leucine may be replaced by phenylalanine (F), valine (V), isoleucine (I), or methionine (M) and make bZIP protein form a variety of binding ways of homodimer and heterodimer. bZIP proteins preferentially combined with ACGT as the core motif to form a palindrome structure (Dröge-Laser et al., 2018).

In plants, *bZIP* TFs play important regulatory roles in plant growth, development, and response to environmental stress. In previous studies, *bZIPs* were found to be expressed in seeds, flowers (Strathmann et al., 2001), leaves (Van Leene et al., 2016), and roots (Ma et al., 2018). Some of them have positive responses to abiotic stress (Zhu et al., 2018) and are highly associated with the abscisic acid (ABA) activation or other metabolic pathways (Lee et al., 2010; Garg et al., 2019). *AtbZIP53* promoted the transcriptional activation of seed maturation genes by forming heterodimers with *bZIP1*, *10*, or *25* (Alonso et al., 2009). *AtbZIP17* was a transcriptional activator involved in salt and stress response. After salt, heat, and ABA treatment, the C-terminal of *AtbZIP17* was cleaved, and then the N-terminal was transferred to the nucleus to activate the expression of salt stress related genes (Zhou et al., 2015). *AtbZIP11* (*GBF6*) actively regulated the expression of the *proline dehydrogenase* (*PRODH*) gene, which participated in amino acid metabolism (Hanson et al., 2008). *AtbZIP11* also induced *TRE1*, *TPP5*, and *TPP6* expression and regulated trehalose metabolism (Ma et al., 2011). *OsbZIP16* found in rice can reduce sensitivity to abiotic stress during the germination of overexpressed seedlings (Pandey et al., 2018). It was found that nuclear-localized C subfamily member *TabZIP6* in wheat (*Triticum aestivum* L.) could not only homodimerize but also form a dimer with other two S subfamily bZIP proteins, which are involved in cold tolerance (Cai et al., 2018). In soybean, *GmbZIP15* negatively regulated *GmWRKY12* and *GmABF1* and made it sensitive to salt and drought stress (Zhang et al., 2020). It was found that *IbbZIP1* in sweet potato was related to salt and drought tolerance and was highly responsive to ABA (Kang et al., 2019).

Licorice is a widely cultivated edible and medicinal crop with high tolerance to stress in the world, which plays very important roles in desertification control, animal husbandry, and human health (Ishimi et al., 2019; Lyu et al., 2020). Unfortunately, the content of most cultivated licorice root is not matching that of wild quality and thus is usually diverted to non-medicinal, food, or confectionary uses (Josef A. Brinckmann, 2020). Furthermore, because of global warming and the decrease of land area suitable for cultivation, there is an urgent need to improve the important traits and quality in licorice breeding. Therefore, it is necessary to make an in-depth study on the development of licorice and its response to environmental factors. The *Glycyrrhiza uralensis* genome was sequenced recently. Although many studies on the different gene families in *G. uralensis* have been reported (Tong et al., 2019; Cui et al., 2020; Goyal et al., 2020; Gao et al., 2021), but the complete genomic information of *G. uralensis* has not been fully explored yet. The comprehensive analysis of *bZIP* gene family in *G. uralensis* has not been reported. In this study, 66 *GubZIP* genes were systematically analyzed, including identification members, phylogenetic relationships, protein structure, conserved domain, duplications in the genome sequence, protein–protein interaction (PPI) network, and related targeted miRNA. The effects of ultraviolet (UV), cadmium (Cd), ABA, methyl jasmonate (MeJA), drought (PEG), and salt (NaCl) on *GubZIP* gene family were investigated. Finally, we performed gene network analyses on the key genes. Comprehensive analysis showed that members of *GubZIP* gene family played an important role in various biological processes and transcriptional regulatory networks of *G. uralensis*. These results lay a foundation for genetic breeding and further study of their biological functions.

## Material and Methods

### Identification of the *GubZIP* Family

The *G. uralensis* data used in this study were derived from the Genome sequence database of *G. uralensis* (http://ngs-data-archive.psc.riken.jp/Gur-genome/download.pl) (Mochida et al., 2017). The hidden Markov model (HMM) profile of the *bZIP* domains (PF00170 and PF07716) was downloaded from the Pfam protein family database (http://pfam.xfam.org) (El-Gebali et al., 2019). The potential *bZIP* genes were identified from the genome of *G. uralensis* using HMMER (E-value < 1 × 10^–5^) (http://hmmer.janelia.org/), and redundant transcripts were removed (Prakash et al., 2017). The nonredundant candidate bZIP protein sequences further confirmed the existence of the bZIP domain by using the National Center for Biotechnology Information (NCBI) Conserved Domain database (https://www.ncbi.nlm.nih.gov/cdd/) and the SMART database (http://smart.embl.de/). The ProtParam tool of the ExPASy (https://web.expasy.org/protparam/) was used to analyze the molecular weight (MW), isoelectric point (pI), instability index (II), aliphatic index (AI), and grand average of hydropathicity (GRAVY) of candidate GubZIPs. The subcellular localization of all predicted GubZIP proteins was analyzed by WoLF PSORT (https://wolfpsort.hgc.jp/) Protein Subcellular Localization Prediction system.

### Phylogenetic Analysis of *GubZIP* Genes

The bZIP protein sequences of *A. thaliana* and *Glycine* max were obtained from Plant Transcription Factor database (http://planttfdb.gao-lab.org/index.php). The full-length bZIP protein sequences of *G. uralensis*, *A. thaliana*, and *G.* max were aligned by ClustalW in MEGAX software using the default parameters (Kumar et al., 2018). An interspecific phylogenetic tree was constructed by the maximum likelihood (ML) method via IQ-TREE software with 1,000 ultrafast bootstrap replications (Nguyen et al., 2015). The tree was further managed by the online tool EvolView v3 (https://www.evolgenius.info/evolview/) (Subramanian et al., 2019).

### Conserved Motifs and Gene Structure Analysis of *GubZIP* Genes

The conserved motifs were analyzed and identified using MEME online (http://meme-suite.org/index.html). In order to clarify the structural diversity of *GubZIP* genes, the intron–exon structures of *GubZIP* genes were visualized using the gene structure display serving GSDS (http://gsds.cbi.pku.edu.cn/) (Hu et al., 2015). The final result was displayed by using TBtools (v1.09832) (Chen et al., 2020).

### Analysis of Cis-regulatory Elements, Location, and Selection Pressure of *GubZIP* Genes

The upstream 1,500-bp regions of the transcriptional start in all candidate *GubZIP* coding sequences (CDSs) (GubZIP64 is less than 1,500 bp in length) were submitted to PlantPAN 3.0 (http://plantpan.itps.ncku.edu.tw/) to identify the regulatory elements (Chow et al., 2019). Based on position information provided by the *G. uralensis* database (http://ngs-data-archive.psc.riken.jp/Gur-genome/download.pl), we visualized the chromosomal distribution of *bZIP* genes in licorice using BioPerl software (Stajich et al., 2002). The similarity of two genes is more than 70%, and the chromosomal distance between the two genes is less than 100 kb, which is defined as tandem replicated gene (Cannon et al., 2004; Audemard et al., 2012). The non-synonymous (ka) and synonymous substitutions (ks) rates were calculated to evaluate the selection pressure. Divergence times (T) were estimated with equation T = Ks/2r × 10^–6^ (Mya) (the r was taken to be 1.5 × 10^–8^ for *G. uralensis*) (Huang et al., 2016). The graph was processed by TBtools (v1.09832) (Chen et al., 2020).

### Prediction of the Interaction Network of GubZIP Proteins and miRNA–*GubZIPs* Interactions

The interaction of GubZIP proteins (orthologs in *A. thaliana*) was performed by STRING software (https://string-db.org/), and the confidence parameter was set to 0.4. In order to predict miRNAs targeting *GubZIP* gene, we queried the full-length CDS of *GubZIP* gene using the psRNATarget service (http://plantgrn.noble.org/psRNATarget/analysis?function=2). To increase stringency, the maximum expectation value was set to 3.0, and the rest used the default parameters. The interaction network of miRNA and *GubZIP* genes was drawn by Cytoscape 3.8.2 software.

### Plant Materials and Stress Treatment

The seeds of *G. uralensis* were treated with 98% sulfuric acid for 30 min to break the seed dormancy, rinsed with sterilized ultra-pure water five times, and then sowed in a seedling plate. Plants grew at 25 C under 60–70% relative humidity in a 16 h day/8 h night cycle in an artificial climate chamber. Four-leaf seedlings were transferred to the hydroponic culture system with 1/2 Murashige–Skoog (MS) liquid medium for different stress treatments. To investigate expression patterns of the genes in response to abiotic stress, PEG6000 (control, 0; drought 1, 10%; drought 2, 20%), NaCl (control, 0; salt 1, 150 mM; salt 2, 300 mM), Cd (control, 0; Cd 1, 0.02 g kg−1; Cd 2, 0.04 g kg−1), UV (control, 0; UV, 2 days, 16-h light/4-h dark/4-h UV (30 W)), MeJA (control, 0; MeJA, 100 μM), and ABA (control, 0; ABA, 100 μM) were used for abiotic stress treatment. The leaves and roots of *G. uralensis* seedlings were collected after 48-h treatment, then quickly frozen in liquid nitrogen, and stored at −80 C. Each treatment consisted of three biological replicates.

### Analysis of the Spatial and Abiotic Stress Expression Patterns of *GubZIP* Genes

In order to analyze the spatial characteristics and differential expression patterns of target genes, the RNA-seq data of leaves and roots (SRP215420) (Li et al., 2020), drought, and salt stress (SRP065514) were downloaded from NCBI (https://www.ncbi.nlm.nih.gov/) and used to determine their expression patterns. The transcript levels of *G. uralensis bZIP* genes were normalized by transcripts per million (TPM).

Total RNA was extracted by RNAprep Pure Plant Kit (TIANGEN, Beijing, China). The concentration and quality of RNA were determined by NanoDrop 2000 spectrophotometer. cDNA was synthesized from total RNA isolated from various tissues by using TaKaRa PrimeScript™ RT Master Mix and gDNA Eraser reverse transcription system, according to the manufacturer’s protocols. Quantitative real-time PCR (qRT-PCR) was performed using QuantStudio six Flex real-time PCR system (Thermo Fisher, Waltham, MA, USA) and TB Green™ Premix Ex Taq™ II (Tli RNaseH Plus) (TaKaRa, Maebashi, Japan). The most stable housekeeping reference gene *GuActin* (accession number GQ404511) was selected as the internal control (Tong et al., 2019). All primers were designed using NCBI Primer Blast website (https://www.ncbi.nlm.nih.gov/tools/primer-blast/). Three independent biological and technical replicates were performed in the qRT-PCR experiments. The relative expression level of GubZIPs was measured by 2−ΔΔCt method, and the final result was visualized by TBtools. In addition, when the relative expression fold changes (treatment/control) ≥2 or ≤0.5 respectively were considered to be differentially upregulated or downregulated. Significance was confirmed by least significant difference (LSD) test (*p* < 0.05). The significant regulatory genes responding to different stresses were analyzed by histogram.

## Results

### Identification and Characterization of *GubZIP* Family Members

A total of 66 members of *GubZIP* gene family were obtained from the genome of *G. uralensis* using a series of bioinformatics methods based on HMM. The candidate *GubZIPs* were identified and named *GubZIPX*, in which X is an integer, representing the ascending order of genes on the corresponding scaffold. *GubZIP34* gene has the shortest protein length with 132 amino acids, whereas GubZIP5 possesses the longest one (788 amino acids). The MW of the proteins ranged from 15.68 kDa (GubZIP34) to 85.39 kDa (GubZIP5) with an average MW of 37.90 kDa. The isoelectric points of the GubZIPs ranged from 4.97 (GubZIP25) to 10.18 (GubZIP44) with an average pI of 7.31. The proteins with an isoelectric point greater than seven accounted for 43.94% of the total number, which means that 29 of the GubZIP proteins were neutral or alkaline. The GRAVY of the 66 sequences had a maximum value of −0.329 (GubZIP43), a minimum value of −1.335 (GubZIP32), and a mean value of −0.75. All negative GRAVY values indicate their hydrophilic nature. The AI of GubZIPs ranged from 46.20 (GubZIP25) to 92.93 (GubZIP60) with an average of 68.20. Their detailed characteristics and subcellular localization are summarized in Table 1.

**TABLE 1:** The detailed characteristics of bZIPs identified in *Glycyrrhiza uralensis.*

Gene name	Protein length (aa)	MW (kDa)	pI	Instability index (II)	Aliphatic index	GRAVY	Stable yes/no^a^	Subcellular location
***GubZIP1***	457	50.66	6.47	43.47	79.02	−0.482	No	nucl
***GubZIP2***	424	46.23	5.91	58.88	57.85	−0.794	No	E.R.
***GubZIP3***	533	59.57	8.61	63.40	62.80	−0.809	No	plas
***GubZIP4***	463	51.14	8.53	56.99	71.97	−0.616	No	nucl
***GubZIP5***	788	85.39	5.74	49.41	62.98	−0.627	No	plas
***GubZIP6***	320	36.13	6.14	66.27	54.94	−0.977	No	plas
***GubZIP7***	472	52.39	8.30	57.34	78.60	−0.511	No	nucl
***GubZIP8***	157	17.65	6.92	52.81	83.82	−0.499	No	cyto
***GubZIP9***	556	60.84	6.48	58.75	58.83	−0.837	No	plas
***GubZIP10***	167	18.85	9.37	64.14	60.24	−0.985	No	plas
***GubZIP11***	144	16.51	6.83	73.60	81.94	−0.678	No	nucl
***GubZIP12***	360	41.15	6.48	52.51	80.81	−0.421	No	nucl
***GubZIP13***	145	16.41	7.87	54.46	86.90	−0.632	No	E.R.
***GubZIP14***	514	54.98	5.91	54.52	53.75	−0.818	No	plas
***GubZIP15***	156	17.38	5.90	50.70	71.41	−0.612	No	nucl
***GubZIP16***	342	36.26	7.68	52.18	52.31	−0.830	No	vacu
***GubZIP17***	215	24.40	6.89	62.61	68.98	−0.989	No	plas
***GubZIP18***	299	32.85	5.11	46.45	68.16	−0.656	No	plas
***GubZIP19***	451	49.38	8.78	52.14	77.69	−0.495	No	mito
***GubZIP20***	557	61.74	6.69	68.37	52.39	−1.009	No	plas
***GubZIP21***	435	46.64	6.82	52.39	58.39	−0.830	No	plas
***GubZIP22***	434	46.71	9.42	43.15	60.94	−0.680	No	nucl
***GubZIP23***	345	37.71	7.15	59.05	62.20	−0.810	No	E.R.
***GubZIP24***	331	35.30	9.07	50.86	48.94	−0.735	No	E.R.
***GubZIP25***	303	31.92	4.97	59.28	46.20	−0.768	No	E.R.
***GubZIP26***	345	38.40	5.87	69.00	69.65	−0.741	No	plas
***GubZIP27***	196	22.49	6.55	56.12	76.07	−0.687	No	vacu
***GubZIP28***	509	57.09	8.88	59.87	73.26	−0.649	No	plas
***GubZIP29***	362	41.20	6.27	57.73	80.11	−0.488	No	nucl
***GubZIP30***	483	53.54	5.93	55.30	76.81	−0.578	No	nucl
***GubZIP31***	666	76.37	8.77	46.34	82.66	−0.567	No	nucl
***GubZIP32***	245	28.15	10.16	44.91	61.67	−1.335	No	E.R.
***GubZIP33***	368	42.38	6.14	67.61	60.16	−1.183	No	plas
***GubZIP34***	132	15.68	10.16	61.21	84.92	−0.559	No	cyto
***GubZIP35***	202	22.82	9.69	54.95	51.14	−1.259	No	vacu
***GubZIP36***	338	37.90	5.03	60.28	83.96	−0.372	No	nucl
***GubZIP37***	328	35.90	6.34	39.51	53.54	−1.107	No	nucl
***GubZIP38***	527	58.76	6.29	66.54	72.47	−0.623	No	plas
***GubZIP39***	221	25.65	5.97	70.18	67.47	−0.985	No	nucl
***GubZIP40***	143	16.27	9.00	52.09	77.83	−0.683	No	nucl
***GubZIP41***	144	16.89	9.13	66.40	83.26	−0.756	No	nucl
***GubZIP42***	170	19.57	9.12	69.61	66.59	−0.712	No	E.R.
***GubZIP43***	347	38.51	7.90	54.06	77.90	−0.329	No	plas
***GubZIP44***	197	22.94	10.18	55.48	83.25	−0.652	No	nucl/cyto
***GubZIP45***	415	45.34	9.79	50.55	61.83	−0.832	No	nucl
***GubZIP46***	324	35.94	8.50	59.78	67.44	−0.810	No	nucl
***GubZIP47***	534	59.10	7.03	72.28	67.17	−0.759	No	plas
***GubZIP48***	504	55.97	6.33	49.14	75.89	−0.497	No	E.R.
***GubZIP49***	386	42.79	9.43	58.25	72.05	−0.721	No	E.R.
***GubZIP50***	404	43.86	6.43	61.02	66.86	−0.683	No	nucl/mito
***GubZIP51***	201	23.15	5.97	77.57	68.36	−0.885	No	plas
***GubZIP52***	241	27.07	7.80	67.50	75.27	−0.868	No	plas
***GubZIP53***	320	33.79	6.70	47.16	63.09	−0.560	No	E.R.
***GubZIP54***	326	36.25	6.10	59.36	69.23	−0.715	No	plas
***GubZIP55***	177	20.99	9.63	67.25	58.42	−1.136	No	E.R.
***GubZIP56***	411	45.14	9.28	59.35	62.82	−0.857	No	plas
***GubZIP57***	382	41.10	5.96	46.34	57.30	−0.769	No	plas
***GubZIP58***	176	19.64	5.37	76.73	58.75	−0.706	No	mito
***GubZIP59***	376	40.44	6.19	54.69	62.87	−0.717	No	E.R.
***GubZIP60***	276	31.00	5.40	60.86	92.93	−0.393	No	nucl
***GubZIP61***	313	34.83	6.62	49.53	69.17	−0.794	No	cyto
***GubZIP62***	165	18.44	9.09	66.27	71.52	−0.940	No	nucl
***GubZIP63***	196	22.51	5.65	61.84	73.11	−0.734	No	E.R.
***GubZIP64***	394	44.90	6.85	57.73	63.17	−0.911	No	nucl/mito
***GubZIP65***	334	36.54	7.08	48.12	65.69	−0.776	No	E.R.
***GubZIP66***	412	44.11	5.78	57.17	53.30	−0.902	No	plas

Note. aa, amino acid; MW, molecular weight; pI, isoelectric point; GRAVY, grand average of hydropathicity; nucl, nucleus; E.R., endoplasmic reticulum; plas, plasma; cyto, cytoplasm; vacu, vacuole; mito, mitochondria.

a A protein whose instability index is smaller than 40 is predicted as stable, while above 40 predicts that the protein may be unstable (Gasteiger et al., 2005).

### Phylogenetic Tree of *bZIP* Genes in *Glycyrrhiza uralensis*, *Glycine max*, and *Arabidopsis thaliana*

**FIGURE 1:**
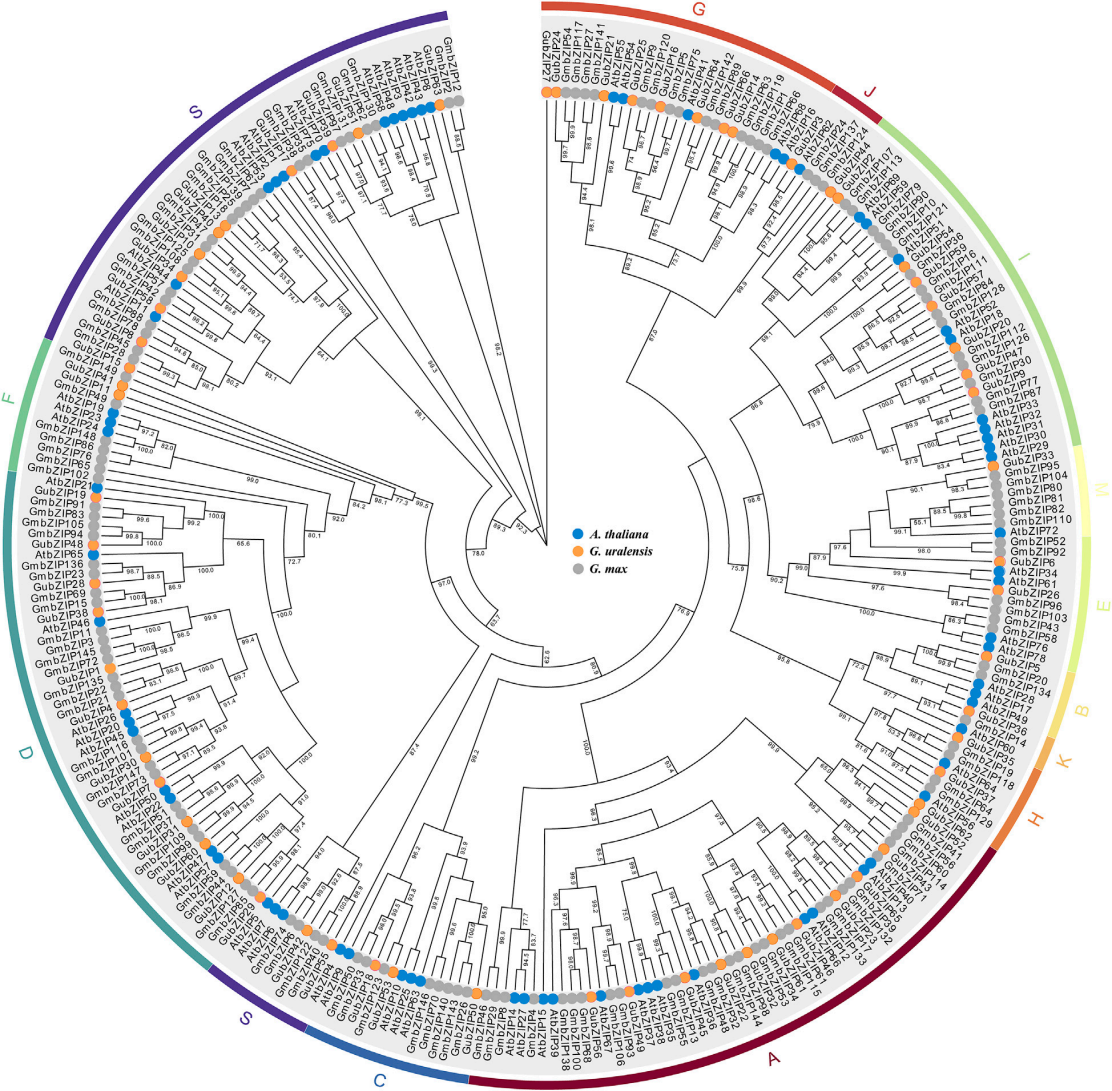
Phylogenetic analysis of bZIP proteins in *Arabidopsis thaliana*, *Glycine max*, and *Glycyrrhiza uralensis*. Protein sequence alignment was carried out in MEGA X by ClustalW, and the evolutionary tree was constructed by maximum likelihood method through IQ-TREE. The pilot value is based on 1,000 duplicates. Only pilot values greater than 50% are displayed. The ends of branches from different species are represented by circles of different colors. The bZIP protein is divided into 13 different evolutionary branches (A–K, M, and S), which are marked by curves with different colors.

To elucidate the evolutionary relationship and classification of the *bZIP* family in different species, we constructed a phylogenetic tree using the entire amino acid of each member from *G. uralensis* (66 *bZIP* genes), model plant *A. thaliana* (74 *bZIP* genes), and cash crop *G.* max (149 *bZIP* genes) (Figure 1) (Dröge-Laser et al., 2018). As shown in Figure 1, the members of the *GubZIP* family were divided into 13 subfamilies (A–K, M, and S) on the basis of the classification of *Arabidopsis*. The S subfamily contains 13 *GubZIP* genes, which is the largest group in the *GubZIP* family, followed by the A and D subfamilies (12 members). The B, J, K, and M subfamilies contain only one *GubZIP* gene, which are the smallest size groups in the *GubZIP* family.

### Gene Structure and Conserved Motif Analysis of *GubZIPs*

**FIGURE 2:**
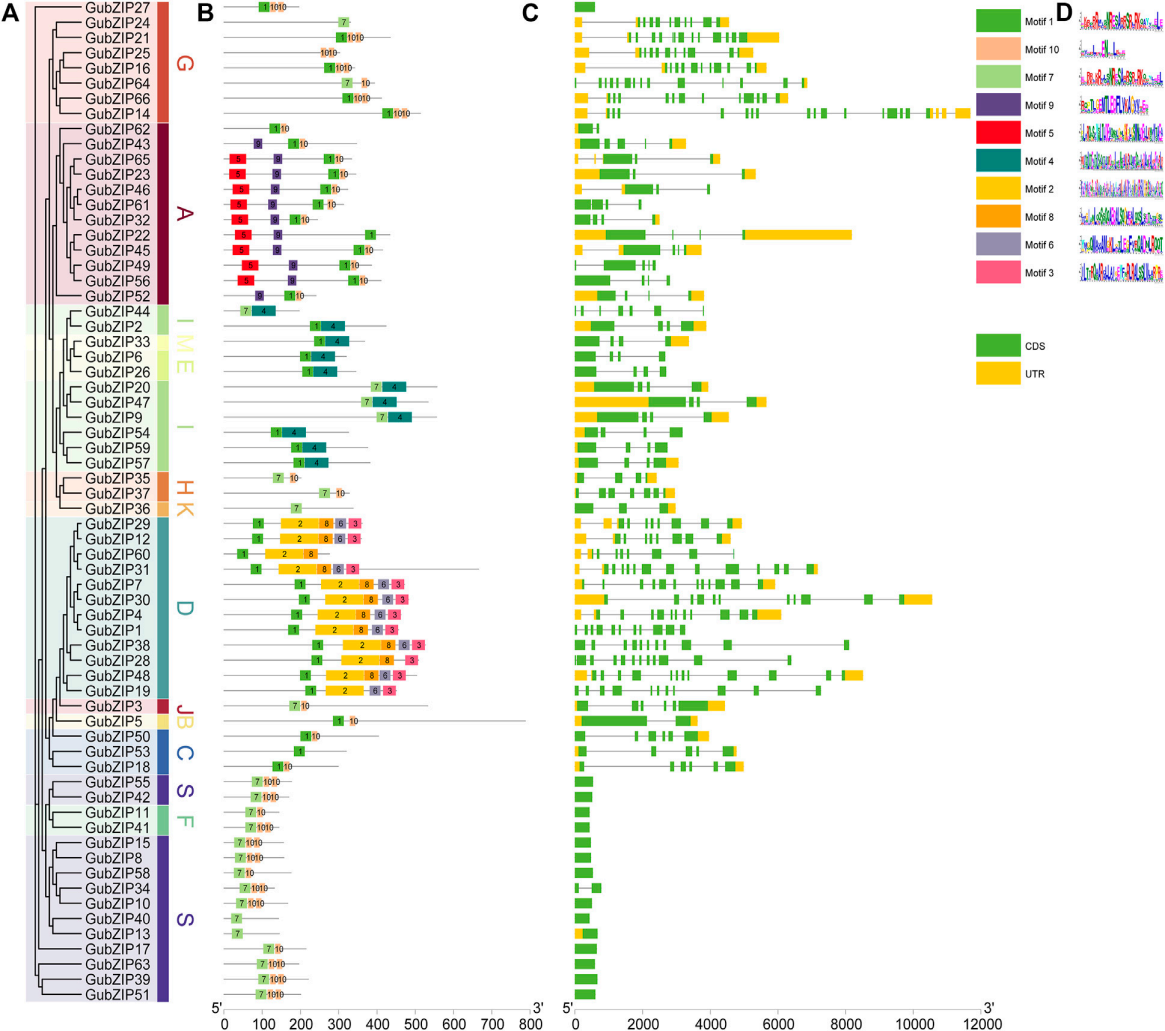
Phylogenetic relationships, gene structure, and architecture of conserved protein motifs in GubZIPs. **(A)** Phylogenetic tree of *GubZIP* genes. **(B)** Distribution of conserved structures in all 66 GubZIP proteins. The colorful boxes delineate different motifs (numbers 1–10). Gray lines represent non-conservative sequences. The protein length can be estimated using the scale at the bottom. **(C)** The exon–intron structure of *GubZIP* genes. The yellow and green boxes represent the untranslated region (UTR) and the coding sequence (CDS), respectively. The gray line indicates the intron. **(D)** Protein motifs in the bZIP members. The colorful boxes delineate different motifs.

To provide greater insight into the gene structure of 66 *bZIP* family genes of *G. uralensis*, a rootless phylogenetic tree of *GubZIPs* was generated (Figure 2A). The conserved motif distribution of GubZIP proteins (Figure 2B) and the exon–intron structure (Figure 2C) were analyzed. The proteins in each subfamily contain the same conserved motifs, which further support the above result of a phylogenetic tree. However, they also have different conserved motifs among various subfamilies. Ten conserved motifs were identified using MEME software (Figure 2D). The length of the motif ranged from 15 to 100 amino acids, and the letter height of the amino acid residue represents its conservation degree. Motif1 has been recognized as a bZIP conserved domain and could be found in most subfamilies except S, K, J, and F, while some subfamilies had unique motif compositions. For example, subfamily A possesses unique motif5 and motif9, whereas motif2, 3, 6, and eight were unique to the subfamily. MEME results showed that GubZIP13, GubZIP24, GubZIP36, and GubZIP40 had only one motif, while the D subfamily contained five motifs except for GubZIP19 and GubZIP60. It was also found that the motif distribution of members in the same subfamily was often highly conservative. For example, most members of the A subfamily had motifs 1, 5, 9, and 10; while most members of the D subfamily had 1, 2, 3, 6, and 8. In addition, the exon–intron structure of the same subfamily members had a similar gene structure. For example, there were almost no introns in the F subfamily and S subfamily, whereas the G subfamily and D subfamily contained a large number of introns and exons. There were significant differences in exon–intron structure among different subfamilies, which further supported the results of the GubZIP phylogenetic tree and classification.

### Chromosomal Scaffold Location and Selection Pressure Analysis of *GubZIP* Genes

**FIGURE 3:**
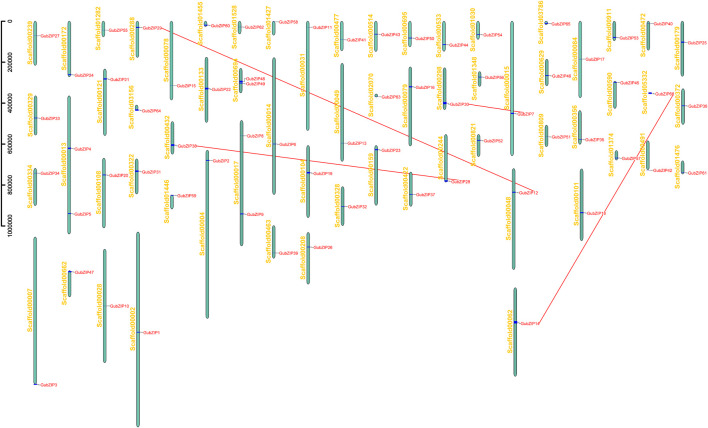
Chromosome scaffold distribution of *GubZIPs*. *GubZIP* genes were located on 63 independent scaffolds. The vertical column represents scaffold, with the number on the left. Pairs of duplicate genes are represented by red dotted lines.

As shown in Figure 3, *bZIP* genes of *G. uralensis* were distributed in 63 separate chromosomal scaffolds. Only scaffold13, 17, and 69 contained two *bZIP* genes, while other scaffolds contained only one *GubZIP*. New cellular functions of genes and their encoded protein products evolve through the mechanism of duplication. Due to the high similarity of retained duplicate genes, a gene is not only regulated by TFs from different families but also bound by multiple members of the same family (Panchy et al., 2016). *GubZIPs* were not evenly distributed across the chromosomal scaffolds. Since the genome of *G. uralensis* was only assembled to the chromosomal scaffold level, we used the manual blast method for duplicate gene finding, only tandem repeat genes were analyzed, and segmental duplication or transposable genes were unable to determine on this level. Then using BioPerl (Stajich et al., 2002), we analyzed the tandem duplication events among the genes. Four tandem repeat gene pairs formed by eight *bZIP* genes were found in *G. uralensis*, of which six members belonged to the D subfamily and the remaining two members belonged to the G subfamily. These lines of evidence suggested that tandem duplication events are the main driving force for the diversity of the *GubZIPs*.

To explore the evolutionary constraints of *GubZIP* genes, the selection pressure of replication gene pairs was analyzed. The average ratios of Ka, Ks, and Ka/Ks of all tandem repeat gene pairs were 0.0991, 0.4303, and 0.2150, respectively (Supplementary Table S3). The Ka/Ks of the four gene pairs (*GubZIP7* and *GubZIP30*, *GubZIP12* and *GubZIP29*, *GubZIP14* and *GubZIP66*, and *GubZIP28* and *GubZIP38*) were all less than 0.5. It was indicated that these gene pairs experienced strong purifying selective pressure. We further used Ks to estimate the time of *GubZIP* genes duplication events during the evolutionary time of the *G. uralensis* genome. The Ks of tandem duplications of *GubZIP* genes occurred from 0.43 (Ks = 0.13) mya to 2.0 (Ks = 0.60) mya, with an average of 1.43 mya.

### Analysis of Cis-regulatory Elements in the *GubZIP* Promoters

**FIGURE 4:**
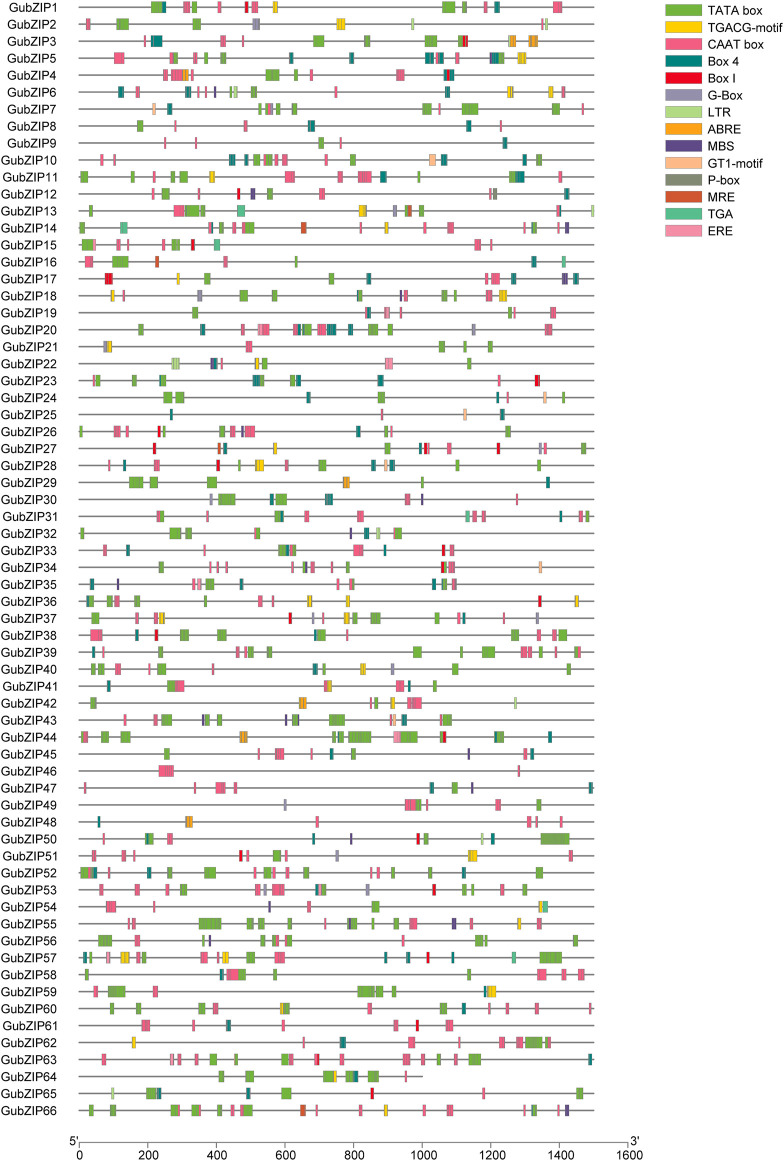
Cis-regulatory element analysis of *GubZIP* gene promoter. The promoter sequence (−1,500 bp) of *GubZIP* genes (−1,000 bp for *GubZIP64*) was inferred on PlantPAN 3.0. The upstream length of each *cis*-regulatory element to the translation start point can be inferred from the scale at the bottom.

TFs regulate the target genes both spatially and temporally through the specific binding of *cis*-regulatory elements (CREs) present in their promoters (Qiu, 2003). In order to explore the CREs of *GubZIP* gene family, the 1.5-kb genomic sequence upstream of each gene was extracted and matched to the PlantPAN 3.0 database. The CREs of the *GubZIPs* are listed in Figure 4. The CREs of *GubZIPs* were involved in transcriptional initiation (transcriptional start, promoter, and enhancer regions), phytohormone responses (ABA, MeJA, gibberellin, ethylene, and auxin response elements), and stress responses (drought, light response, and low temperature). Several CREs were identified to be involved in the hormonal response, such as MeJA (TGACG-motif), auxin (TGA) response elements, and gibberellin response element (P-box). At the same time, stress-response elements related to ABA (ABRE), low-temperature reactivity (LTR), ethylene (ERE) responses, and the MYB binding site (MBS) involved in drought induction were also identified. ABA response elements were detected in *GubZIP29*, *3*, *4*, *42*, *44*, and *48*, which belonged to subfamilies J, D, S, and I. The MYB binding site (MBS) involved in drought induction was detected in 20 members. Moreover, a total of 18 CREs with light-responsive components were identified. MYB binding sites (MRE) involved in light response, light-responsive elements (GT1-motif), *cis*-acting regulatory elements about light responsiveness (G-Box), part of a conserved DNA module involved in light responsiveness (Box4), and light-responsive elements (Box I) were detected in five, nine, 13, 59, and 25 members, respectively. The results showed that *GubZIP* genes may play important roles in plant growth and stress responses, and especially in the response to light.

### Protein–Protein Interaction Network of GubZIPs

**FIGURE 5:**
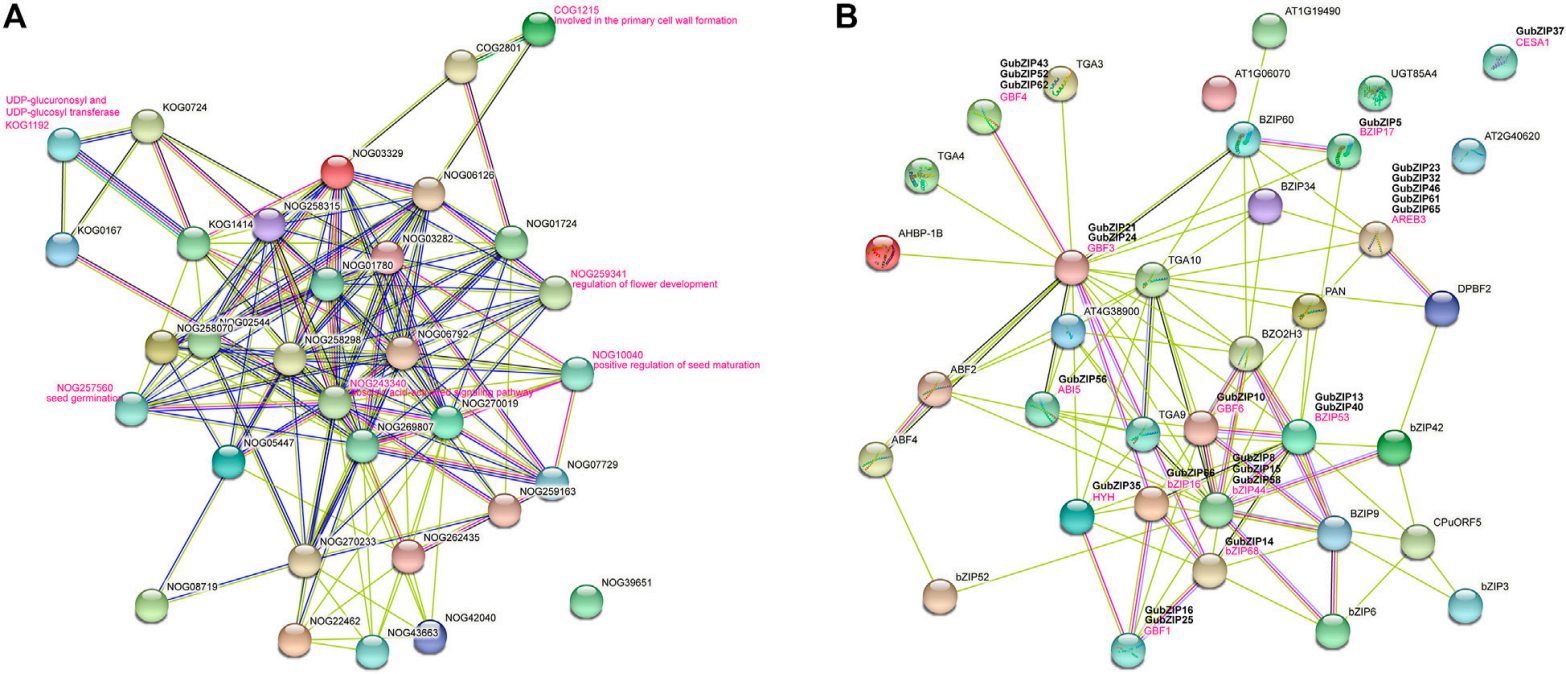
Interaction network of GubZIP proteins. **(A)** Search for the orthologs in *Arabidopsis* matching GubZIPs. **(B)** The GubZIP protein–protein interaction network. This network was predicted by online software STRING. The GubZIP proteins related to abiotic stresses are shown in the bold black font above the *Arabidopsis* orthologs.

Based on the studied *AtbZIPs* and the orthologs in *Arabidopsis*, we could speculate about the functions of most *GubZIP* genes. To analyze them, we constructed an interaction network analysis by using the STRING database based on search for protein families (Figure 5A) and multiple sequences (Figure 5B). Through the GubZIP protein family interaction network, it was found that there were 14 members involved in the ABA activation signal pathway (NOG243340), of which 10 members belonged to the A subfamily (83.3% of the A subfamily), three belonged to the S subfamily (23.1% of the S subfamily), and one belonged to the G subfamily (12.5% of the G subfamily). The members involved in primary cell wall formation (COG1215), UDP-glycosyltransferase (KOG1192), flower development regulation (NOG259341), and positive regulation of seed maturation (NOG10040) were from H, G, D, and C subfamilies. The members involved in seed germination (NOG257560) were GubZIP49 and GubZIP56, both belonging to the A subfamily. The results suggested that the interaction network of the *GubZIP* family was spread around the ABA signal pathway, and their regulation network plays an important role in the development of *G. uralensis*. Wang et al. showed a similar phenomenon in *GHbZIPs* (Wang et al., 2020). As shown in Figure 5B, bZIP53 (GubZIP13 and 40) interacted directly with BZIP17 (GubZIP15), GBF3 (GubZIP21 and 24), ABI5 (GubZIP56), GBF6 (GubZIP10), HYH (GubZIP35), bZIP16 (GubZIP66), bZIP44 (GubZIP 8, 15, and 58), and bZIP68 (GubZIP14). It was suggested that bZIP53 is involved in cellular response to abiotic stress response and positive regulation of seed maturation (Alonso et al., 2009). The direct interactions of bZIP68 (GubZIP14) and HYH (GubZIP35) with GBF1 (GubZIP16 and 25), GBF4 (GubZIP43, 52, and 62) with GBF3 (GubZIP21 and 24), and AREB3 (GubZIP23, 32, 46, 61, and 65) with GBF6 (GubZIP10) were found in PPI network. The interaction between GBF4 (homolog of GubZIP43, 52, and 62) and bZIP68 (homolog of GubZIP14) was regulated by light or other hormones (Terzaghi et al., 1997).

### Analysis and Prediction of miRNAs Associated With *GubZIPs*

**FIGURE 6:**
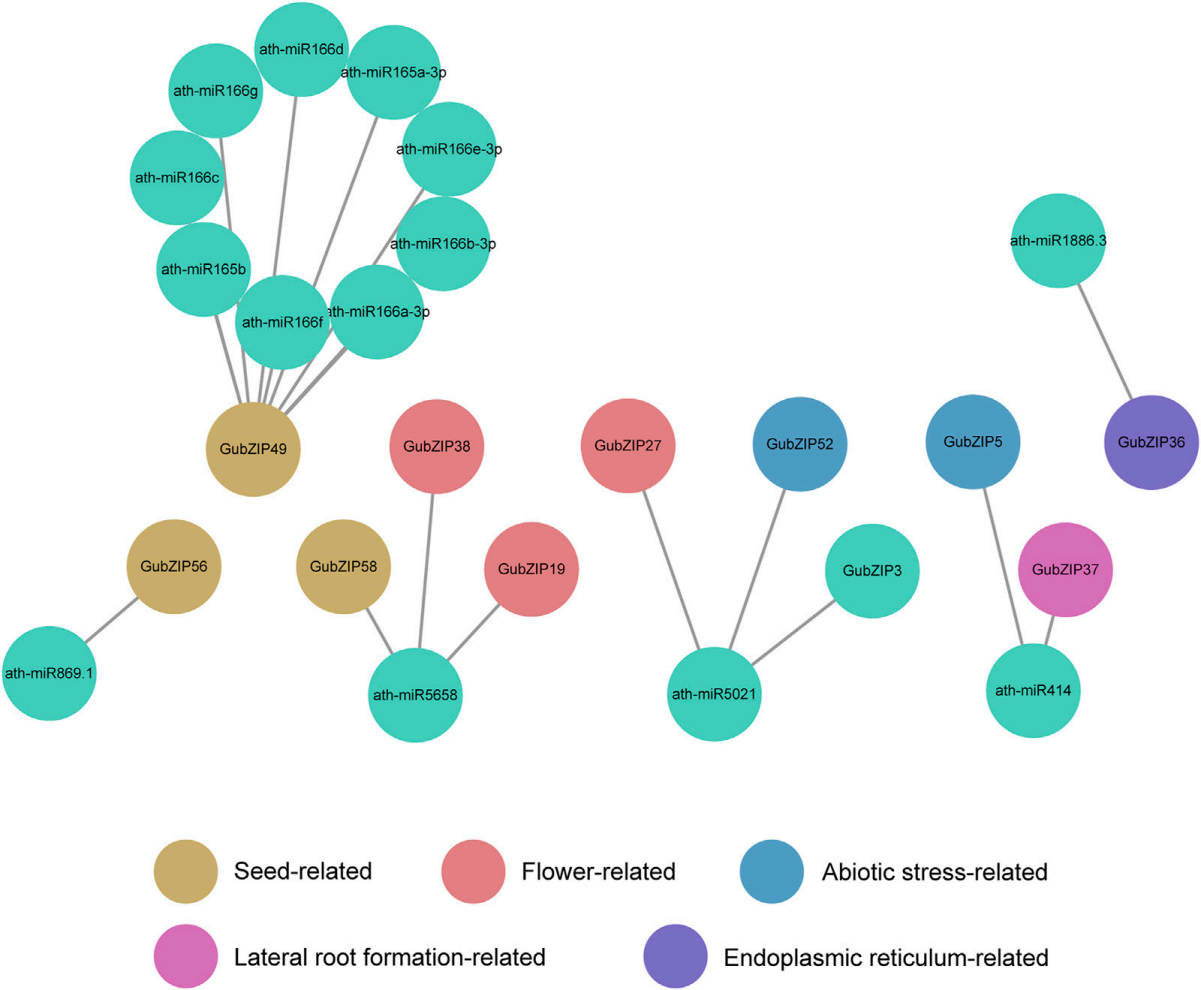
Interaction network of miRNAs and their targeted *GubZIPs* using Cytoscape software.

With the use of the psRNATarget server, the miRNAs associated with *GubZIP* genes were predicted based on annotated data of *A. thaliana*. The results showed that six miRNA families were identified. The targeted *GubZIPs* (11 members) belonged to K, B, D, H, and A subfamilies (Figure 6), which were involved in the development, abiotic stress, lateral root formation, and endoplasmic reticulum process. The results showed that not only one miRNA family targeted unique *GubZIP*, but also one miRNA family could target multiple *GubZIPs* with different functions. It is suggested that miRNA plays a potential role in the PPI relationship of GubZIPs. The complicated regulatory relationship of miRNAs and multi-functional *GubZIPs* provided the opportunity for the collaboration of genes with different functions.

### Analysis and Verification of Spatial and Abiotic Stress Expression Patterns of *GubZIP* Genes

**FIGURE 7:**
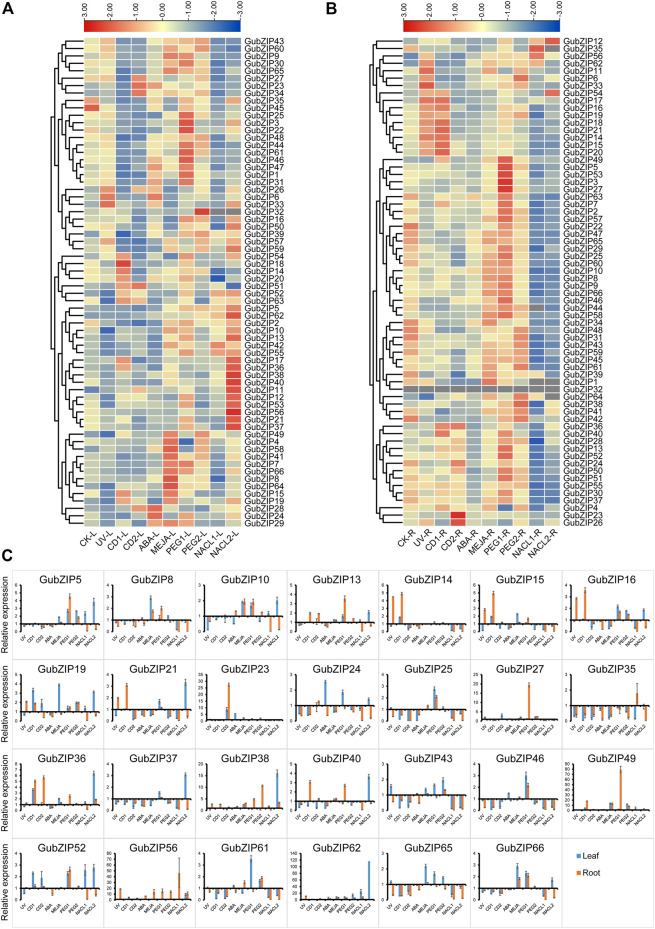
Expression pattern of *bZIP* genes in *Glycyrrhiza uralensis* under abiotic stress. **(A)** Heat maps of the relative expression of *bZIP* genes in leaf tissue of *G. uralensis* under abiotic stress. **(B)** Heat maps of the relative expression of *bZIP* genes in root tissue of *G. uralensis* under abiotic stress. The level of expression is indicated by the graded color code. **(C)** The qRT-PCR expression profile of *GubZIP* genes under abiotic stress. The expression level of *GubZIPs* in the control group was standardized as “1.” The vertical bar represents the standard error of the average. Significance is indicated on the bar graph by a–i for the difference between gene expression; significance was confirmed by least significant difference (LSD) test (*p* < 0.05).

To verify the expression patterns of *GubZIP* family members, the RNA-seq data of leaves and roots under drought and salt stress were accessed in NCBI. *GubZIP* genes showed different expression patterns under different spatial and abiotic stresses (Supplementary Figure S1). *GubZIP* members were clustered into three categories in different tissues (Supplementary Figure S1A). One group was highly expressed in roots containing 31 members (46.97%) in total, while 23 members (34.85%) were specifically expressed in leaves. The rest of the members were expressed in both leaves and roots. In the abiotic stress expression (Supplementary Figure S1), *GubZIP* members showed various expression patterns under different stress conditions. For example, *GubZIP40* was downregulated under salt stress but upregulated under drought stress; *GubZIP6*, *9*, *33*, *54*, and *57* were upregulated under salt stress but downregulated under drought stress. Different expression patterns reflected the different roles of *GubZIP* genes in the corresponding pathways, which provide a reference for the identification of functional genes. In order to further study the biological function of *GubZIP* genes, qRT-PCR was used to analyze the expression patterns of *GubZIP* genes under various abiotic stresses condition. As shown in Figure 7, the expression pattern of *GubZIPs* under salt and drought stress was similar to that of RNA-seq data.

There were eight and 56 *GubZIP* genes that were differentially expressed in roots and leaves under salt stress, respectively. *GubZIP12*, *17*, *35*, *56*, and *62* were upregulated in roots; *GubZIP5*, *11*, *28*, and *62* were upregulated in leaves in low salt concentration levels; *GubZIP6*, *12*, *38*, *54*, and *56* were upregulated in roots; and 26 genes were upregulated in leaves under low salt concentration stress condition. In roots, the expression level of *GubZIP12* increased with the increasing NaCl concentration, whereas the expression of *GubZIP56* was upregulated compared with control but decreased with the increasing NaCl concentration. The expression of *GubZIP17*, *35*, and *62* was upregulated from low salt concentration but decreased at high salt concentration. On the contrary, the expression of *GubZIP6*, *38*, and *54* was upregulated in high salt concentration. In leaves, the expression levels of *GubZIP3*, *21*, *26*, *37*, and *56* were downregulated at low salt concentration but upregulated at high salt concentration. The expression of *GubZIP62* was upregulated in both roots and leaves at low salt concentration.

There were 35 and 41 *GubZIP* genes that were differentially expressed in roots and leaves, respectively, under drought stress. A total of 27 *GubZIP* genes were upregulated, four *GubZIP* genes (*GubZIP4*, *11*, *26*, and *34*) were downregulated in roots under low concentration drought stress, 25 *GubZIP* genes were upregulated, and only *GubZIP4* was downregulated in leaves. Ten *GubZIP* genes were upregulated in roots, three *GubZIP* genes (*GubZIP21*, *35*, and *63*) were downregulated in roots under high concentration drought stress, and 14 genes were upregulated and 10 GubZIP genes were downregulated in leaves. In roots, the expression level of *GubZIP6* and *38* increased with the increasing drought stress concentration, while the expression of *GubZIP27* and *49* increased compared with that of the control but decreased with the increasing PEG concentration. The expression level of *GubZIP11* changed from downregulation under low drought stress to upregulation under high drought stress. In leaves, the expression level of *GubZIP3* changed from upregulated under low drought stress to downregulated under high drought stress. And the expression of *GubZIP62* was upregulated in roots and leaves under low concentration drought stress.

*GubZIP* genes were differentially expressed in roots (16 *GubZIPs*) and leaves (34 *GubZIPs*) under the MeJA treatment condition. There were 10 and 19 *GubZIP* genes upregulated in roots and leaves, while six and 14 genes were downregulated, respectively. The expression of *GubZIP6*, *33*, and *56* was upregulated in roots but downregulated in leaves, whereas *GubZIP42* showed the opposite expression pattern. The expression of *GubZIP11*, *38*, *49*, *58*, *62*, and *64* was upregulated in roots and leaves, whereas the expression of *GubZIP16* and *35* was downregulated in roots and leaves.

There were 30 and 25 *GubZIP* genes differentially expressed in roots and leaves, respectively, under ABA treatment conditions. Furthermore, the expression levels of *GubZIP18*, *22*, *36*, *37*, and *42* were downregulated in both roots and leaves. *GubZIP24* and *31* showed a downregulated pattern in roots and upregulated levels in leaves, whereas *GubZIP56* showed opposite expression tendency in roots and leaves. The expression level of *GubZIP62* was upregulated in both roots and leaves.

There were 45 and 51 differentially expressed genes in roots and leaves, respectively, under cadmium stress conditions. Fourteen *GubZIP* genes were upregulated and 24 *GubZIP* genes were downregulated in roots under low cadmium concentration, while nine *GubZIP* genes were upregulated and 29 *GubZIP* genes were downregulated in leaves. *GubZIP23*, *26*, *36*, and *56* were upregulated in roots, while 30 of *GubZIP* genes were downregulated in high cadmium concentration. In roots, the expression of *GubZIP56* was upregulated when compared with that of the control, but it decreased with the increase of cadmium concentration. The expression levels of *GubZIP23* and *26* were downregulated under low cadmium stress condition but upregulated under high-stress conditions, while *GubZIP12*, *17*, and *18* showed opposite expression patterns. In leaves, the expression levels of *GubZIP11* and *51* increased with the increase in cadmium concentration, while the expression levels of *GubZIP17* and *18* were upregulated under low cadmium stress conditions but downregulated under high concentration. *GubZIP23*, *26*, *27*, and *34* showed a downregulated expression pattern under low cadmium stress but upregulated under high cadmium stress. The expression levels of *GubZIP1*, *3*, *6*, *22*, *31*, *35*, *41*, *48*, *59*, *60*, and *65* were downregulated in roots and leaves. Interestingly, the expression of *GubZIP26* was downregulated at low cadmium concentration but upregulated at high cadmium concentration in both roots and leaves.

There were 36 and 23 differentially expressed genes in roots and leaves, respectively, under UV stress conditions. The expression levels of *GubZIP22*, *24*, *35*, *42*, and *63* were downregulated in both roots and leaves; while *GubZIP6*, *11*, *26*, *33*, and *54* showed opposite expression patterns in roots and leaves. The expression level of *GubZIP39* was downregulated in roots and upregulated in leaves. The expression levels of *GubZIP12*, *15*, *18*, *21*, *38*, and *56* were upregulated in roots but downregulated in leaves.

The results revealed the different response mechanisms of *GubZIPs* under abiotic stress. The function of *GubZIP* genes can be more effectively estimated in the future.

## Discussion

The *bZIP* TFs exist widely in the plant kingdom and play an important role in plant growth, development, and the response to corresponding environmental changes (Herath and Verchot, 2021). bZIP proteins have been identified in many plant species, including *A. thaliana* (Dröge-Laser et al., 2018), soybean (Zhang et al., 2018), poplar (Zhao et al., 2021), and *Vitis vinifera* (Liu et al., 2014). However, up to now, no systematic study on *bZIP* gene family of *G. uralensis* has been reported. The genome-wide analysis of *GubZIPs* would aid in their further functional analyses as well as licorice breeding research. In this study, a total of 66 *bZIP* genes of *G. uralensis* were identified in the genome. The genes belonging to the same subfamily in the phylogenetic tree had a relatively conservative relationship, and *GubZIPs* were divided into 13 subfamilies, which is consistent with *A. thaliana*. The stability of GubZIP protein was estimated by II (Table 1). A protein whose II is smaller than 40 was predicted as stable. The II values for 66 GubZIP proteins were all above 40, predicted as unstable. The AI of the protein was used to evaluate the thermal stability of GubZIP protein (Gasteiger et al., 2005). In this study, the AI of D and S subfamily proteins was generally higher than that of G subfamily proteins, indicating that D and S subfamily GubZIP proteins have higher thermal stability than G subfamily proteins. The prediction results of subcellular localization of GubZIP showed that subfamilies with multiple members would not be distributed on the same subcellular structure. In eukaryote cells, members of the multigene family were localized to specific subcellular compartments, suggesting phylogenetic divergence and distinct functional roles *in vivo* (Vierling, 1991).

Tandem replication is an evolutionary process whereby a segment of DNA was replicated and proximally inserted. It was considered the main driving force that expands gene families, which is a key driver of adaptive evolution in species that are currently facing widespread environmental challenges (Song et al., 2019). Comparative genomic analysis between closely related species has revealed that tandem duplication is one of the major mechanisms creating new genes, particularly genes clustered into a gene family, which have been documented in many organisms (Anderson and Roth, 1977; Hazkani-Covo and Graur, 2007; Fan et al., 2008). *bZIP* gene family, as one of the largest known TF families in plants, has a large number of subfamilies and members (Liu and Chu, 2015). However, the Ka/Ks ratios of *GubZIP* tandem repeat gene pairs were all lower than 0.5, which indicated that these repetitive *GubZIP* genes may be affected by strong purification selection and maintain a relatively conservative function in different species. For example, as tandem repeat pairs, *GubZIP14* and *66* were upregulated in the root under UV and hormone treatment conditions, respectively, which showed a highly similar expression profile to the regulation of homologous in *A. thaliana* (*bZIP68* and *bZIP16*) by light-induced or hormone-controlled stimuli (Shen et al., 2008). Repetitive genes also provide templates for new genes, which have new functions (Fan et al., 2008).

CREs play critical roles in the regulation of plant stress responses. ABA-responsive elements (ABREs) are involved in the plant response to ABA hormone treatment, drought, and salt stress. LTR responds to low-temperature reactivity. The TCA and ERE element are correlated with the expression level of MeJA and ethylene. There were 14 members of *GubZIPs* involved in the ABA-activated signaling pathway (NOG243340), which *cis*-acting regulatory elements associated with light, low temperature, drought, ethylene, and MeJA located in their upstream sequences of promoters. Several *cis*-acting elements responding to light and MeJA were found in the promoter regions of *GubZIP37* that were involved in primary cell wall formation (COG1215). The expression of *GubZIP37* was downregulated under cadmium, ABA, MeJA, and salt stress conditions, suggesting that the cell damage caused by stress may be due to the effect of related *cis*-acting regulatory elements. The upstream sequences of *GubZIP1* and *64* that were involved in the regulation of flower development (NOG259341) and UDP-glycosyltransferase (KOG1192) contain several *cis*-acting elements related to light response. The expression level of *GubZIP64* was downregulated under UV stress and upregulated under MeJA and low concentration drought stress, which was consistent with previous studies that moderate drought could increase the content of secondary metabolites in *G. uralensis* (Li et al., 2011). A great deal of *cis*-elements responding to environmental stress were found in the promoter regions of the *GubZIP50*, such as ERE (ethylene response), MRE (metal-responsive element), and LTR (low-temperature reactivity). *GubZIP50* was downregulated in leaves under a high concentration of cadmium treatment, which may indicate that *GubZIP50* was involved in the response to heavy metal tress that may be related to the decrease of mitotic index caused by cytotoxicity (Chidambaram et al., 2009). The analysis of *cis*-acting elements and the prediction of the protein interaction network of 66 *GubZIP* family members indicated the enrichment of *cis*-acting elements, such as ABRE, ERE, Box 4, GT 1-motif, or G-Box; and the interaction network together with homologs in *Arabidopsis* suggested that *GubZIP* family members also play wide roles in the light response, hormone response, and growth and development of licorice plants.

Gene expression is regulated by *cis*-acting elements of the upstream promoter, which are sites involved in transcriptional initiation and regulating the specific binding of proteins and often determine the transcriptional process of gene expression (Zou et al., 2011; Hernandez-Garcia and Finer, 2014). As shown in Supplementary Figure S1, the expression trend of *GubZIP* TFs showed different profiles under abiotic stress, which were based on RNA-seq data (from public databases). The results of qRT-PCR verification (Figure 7) showed the expression profiles of *bZIPs* in leaves and roots of *G. uralensis* under various abiotic stresses. The expression pattern of *GubZIPs* under salt and drought stress treatments was similar to that of RNA-seq data. *GubZIP11* belongs to the F subfamily. In root tissues, Cd, NaCl, and low drought (PEG) stress can downregulate its expression, while MeJA and UV can upregulate its expression. In leaf tissue, the expression of six kinds of abiotic stress was upregulated. He et al. proved that soybean *GmbZIP19* was positively regulated by ABA, jasmonic acid (JA), and salicylic acid (SA) and negatively regulated by salt and drought, showing tolerance to plant pathogens (He et al., 2020). The phylogenetic tree showed that *GubZIP11* was highly homologous *GmbZIP19*, which belongs to the F subfamily. This suggested that *GubZIP11* may have a function similar to that of *GmbZIP19* in improving the tolerance of pathogens.

Some members of the S subfamily in licorice showed specific expression patterns under salt and drought stress and contained a MeJA response element (TGACG-motif) in the promoter region. Yang et al. reported that the S subfamily *GmbZIP2* may enhance drought and salt resistance by regulating reactive oxygen species (ROS) in soybean (*G. max*) (Yang et al., 2020). For example, *GubZIP42* and *55*, the homologs of *GmbZIP2*, were upregulated in leaves and downregulated in roots under salt stress. In addition, both of them had TGACG-motif, which suggested that they could improve their tolerance to salt stress via the process of hormone regulation. It suggested that *GubZIPs* of the S subfamily may be responsible for the resistance to salt and drought stress.

Li et al. reported that *CabZIP25* (member of subfamily A in *Capsicum annuum*) not only can maintain the stability of chlorophyll in pepper (*C. annuum*) to enhance salt tolerance but also can increase the germination rate, fresh weight, and root length of overexpressed *A. thaliana* (Gai et al., 2020). Gai et al. have also proved that *NtbZIP62*, which belongs to subfamily A in tobacco (*N. tabacum*), can be induced by salt and ABA to enhance its salt tolerance (Li et al., 2021, 62). *GubZIP52* and *62*, which belong to the same A subfamily as *CabZIP25* and *NtbZIP62*, were also highly expressed under low salt concentration and ABA treatment, so their high expression may have a positive effect on the salt tolerance of licorice. In the present study, almost all members of the A subfamily in *G. uralensis* were homologous to *ABI5* and *AREB3* in *A. thaliana* (Figure 5B), involved in the ABA signaling process. For example, *GubZIP56* and *62* were upregulated in root under the treatment of ABA, MeJA, NaCl, and PEG, whereas they were downregulated in leaf under the treatment of ABA and MeJA and then were upregulated at a later time. All the results suggested that members of subfamily A in licorice may also play wide roles in ABA response.

The *cis*-acting elements analysis also showed that both *GubZIP16* and *25* (belonged to the G subfamily) had light-regulated elements. The expressions of *GubZIP16* and *25* were upregulated and downregulated respectively under UV treatment, while they showed a downregulated pattern under NaCl treatment in roots. It has been reported that *GBF1*, the homolog in *A. thaliana*, was a negative regulator of blue light-dependent hypocotyl expansion (Gangappa et al., 2013) and can trigger ROS accumulation (Giri et al., 2017, 1). *ChbZIP1* that belongs to the G subfamily may enhance antioxidation by regulating genes related to oxidant detoxification in *Alkaliphilic Microalgae Chlorella* to adapt to abiotic stress (Qu et al., 2021). These results suggested that the G subfamily of *GubZIPs* may respond to pathogen invasion and environmental stress factors by regulating the accumulation of ROS.

## Conclusion

The *bZIP* gene family plays an important role in plant growth, development, and response to biotic and abiotic stress. We undertook a comprehensive genome-wide characterization and expression analysis of *bZIP* gene family in licorice under different abiotic stresses. A total of 66 *GubZIP* genes were identified and classified into 13 subfamilies. Proteins within the same subfamilies contained very similar gene structures and protein motifs. We detected a large number of tandem duplication events, which suggested that tandem duplication events were the main driving force for the evolution of *bZIP* gene family in licorice. The expression patterns of the *GubZIP* family were verified by heat map and qRT-PCR. It was showed that certain genes were significantly upregulated or downregulated under abiotic stresses. Gene expression patterns can provide important clues for gene function. It was found that *GubZIP11*, an ortholog of *GmbZIP19*, showed specific response to MeJA and UV treatments in root tissue, suggesting that it might be a candidate gene to improve the tolerance to pathogens in licorice. And our findings also indicated that several genes (such as *GubZIP56*, *GubZIP62*, *GubZIP64*, and *GubZIP42*) played key roles in abiotic stress tolerance. The comprehensive understandings of *GubZIP* gene family provide useful information for further functional studies to elucidate their regulation mechanism and lay the foundation for cultivating high-quality cultivars of *G. uralensis* through molecular breeding methods in the future.

## Data Availability

The datasets presented in this study can be found in online repositories. The names of the repository/repositories and accession number(s) can be found in the article/Supplementary Material.
