# Supplementary material for: Genome-Wide Characterization and Expression Analysis of bZIP Gene Family Under Abiotic Stress in Glycyrrhiza uralensis
Source: Front Genet. 2021 Oct 5;12:754237. doi: 10.3389/fgene.2021.754237 (PMC8525656; doi:10.3389/fgene.2021.754237)
Supplement: Supplementary file 1 [file DataSheet1.docx]

Supplementary Material

# Supplementary Tables

**Supplementary Table 1 |** Putative cis-acting element sequences in promoter regions of *GubZIP* genes.

| Classification | cis-element | Sequence | Description |
| --- | --- | --- | --- |
| Transcription initiation | TATA box | TAATA | core promoter element around -30 of transcription start |
|  | CAAT box | CAATT | common cis-acting element in promoter and enhancer regions |
| Phytohormone responsiveness | ABRE | ACGTGGC | cis-acting element involved in the abscisic acid responsiveness |
|  | TGACG-motif | TGACG | cis-Acting regulatory element was about the MeJA-responsiveness |
|  | ERE | ATTTCAAA | Ethylene-responsive element |
|  | TGA | AACGAC | Auxin-responsive element |
|  | P-box | CCTTTTG | Gibberellin-responsive element |
| Stress responsiveness | MBS | TAACTG | MYB binding site involved in drought-inducibility |
|  | MRE | AACCTAA | MYB binding site involved in light responsiveness |
|  | GT1-motif | GGTTAAT | Light responsive element |
|  | G-Box | CACGTT | cis-Acting regulatory element was about light responsiveness |
|  | Box 4 | ATTAAT | Part of a conserved DNA module involved in light responsiveness |
|  | Box I | TTTCAAA | light responsive element |
|  | LTR | CCGAAA | cis-acting element involved in low-temperature responsiveness |

**Supplementary Table 2 |** Details for predicted miRNA targeting *GubZIP* genes.

| miRNA_Accession | Target Name | Expectation | miRNA start | miRNA end | Target start | Target end | miRNA_aligned_fragment | Target_aligned_fragment |
| --- | --- | --- | --- | --- | --- | --- | --- | --- |
| *ath-miR5658* | *GubZIP58* | 0 | 1 | 21 | 335 | 355 | AUGAUGAUGAUGAUGAUGAAA | AAUCAUCAUCAUCAUCAUCAU |
| *ath-miR5021* | *GubZIP3* | 1.5 | 1 | 20 | 55 | 74 | UGAGAAGAAGAAGAAGAAAA | UCUUCUUCUUCUUCUUCUUA |
| *ath-miR5021* | *GubZIP27* | 1.5 | 1 | 20 | 148 | 167 | UGAGAAGAAGAAGAAGAAAA | CUUUCUUCUUCUUCUUCUUC |
| *ath-miR165a-3p* | *GubZIP49* | 2.5 | 1 | 21 | 414 | 434 | UCGGACCAGGCUUCAUCCCCC | UGUGGAUGAAGUUUGGUCUGA |
| *ath-miR165b* | *GubZIP49* | 2.5 | 1 | 21 | 414 | 434 | UCGGACCAGGCUUCAUCCCCC | UGUGGAUGAAGUUUGGUCUGA |
| *ath-miR414* | *GubZIP5* | 2.5 | 1 | 21 | 837 | 857 | UCAUCUUCAUCAUCAUCGUCA | UUCUGAUGGUGAUGAGGAUGA |
| *ath-miR5021* | *GubZIP52* | 2.5 | 1 | 20 | 55 | 74 | UGAGAAGAAGAAGAAGAAAA | AUAUCUUCUUCUUCUUCUUC |
| *ath-miR5658* | *GubZIP38* | 2.5 | 1 | 21 | 187 | 207 | AUGAUGAUGAUGAUGAUGAAA | CUCCAUCAUAAUCAUCAUCAU |
| *ath-miR869.1* | *GubZIP56* | 2.5 | 1 | 21 | 1058 | 1078 | AUUGGUUCAAUUCUGGUGUUG | UAGAAGCAGAAUUGAACCAAU |
| *ath-miR166a-3p* | *GubZIP49* | 3 | 1 | 21 | 414 | 434 | UCGGACCAGGCUUCAUUCCCC | UGUGGAUGAAGUUUGGUCUGA |
| *ath-miR166b-3p* | *GubZIP49* | 3 | 1 | 21 | 414 | 434 | UCGGACCAGGCUUCAUUCCCC | UGUGGAUGAAGUUUGGUCUGA |
| *ath-miR166c* | *GubZIP49* | 3 | 1 | 21 | 414 | 434 | UCGGACCAGGCUUCAUUCCCC | UGUGGAUGAAGUUUGGUCUGA |
| *ath-miR166d* | *GubZIP49* | 3 | 1 | 21 | 414 | 434 | UCGGACCAGGCUUCAUUCCCC | UGUGGAUGAAGUUUGGUCUGA |
| *ath-miR166e-3p* | *GubZIP49* | 3 | 1 | 21 | 414 | 434 | UCGGACCAGGCUUCAUUCCCC | UGUGGAUGAAGUUUGGUCUGA |
| *ath-miR166f* | *GubZIP49* | 3 | 1 | 21 | 414 | 434 | UCGGACCAGGCUUCAUUCCCC | UGUGGAUGAAGUUUGGUCUGA |
| *ath-miR166g* | *GubZIP49* | 3 | 1 | 21 | 414 | 434 | UCGGACCAGGCUUCAUUCCCC | UGUGGAUGAAGUUUGGUCUGA |
| *ath-miR1886.3* | *GubZIP36* | 3 | 1 | 21 | 982 | 1002 | AAUUAAAGAUUUCAUCUUACU | ACGAAGAUGAAAUUUUUAUUU |
| *ath-miR414* | *GubZIP37* | 3 | 1 | 21 | 303 | 323 | UCAUCUUCAUCAUCAUCGUCA | UGAUGAUAUUGAUGAAGAUGA |
| *ath-miR5658* | *GubZIP19* | 3 | 1 | 21 | 469 | 489 | AUGAUGAUGAUGAUGAUGAAA | UCAGAUCAUCAUCAUCAUCAG |

**Supplementary Table 3 |** Selection pressure and divergence time for gene tandem repeats with *Ka* and *KS* values.

| Syntenic gene pairs | Method | *Ka* | *Ks* | *Ka/Ks* | P-Value(Fisher) | Divergence-Time（MYA） |
| --- | --- | --- | --- | --- | --- | --- |
| *GubZIP7* & *GubZIP30* | MA | 0.117037 | 0.500904 | 0.233652 | 2.82771E-33 | 1.66968 |
| *GubZIP12* & *GubZIP29* | MA | 0.163159 | 0.602094 | 0.270985 | 2.02942E-26 | 2.00698 |
| *GubZIP14* & *GubZIP66* | MA | 0.0205931 | 0.129268 | 0.159306 | 1.18193E-11 | 0.43089 |
| *GubZIP28* & *GubZIP38* | MA | 0.0957769 | 0.488845 | 0.195925 | 8.8467E-39 | 1.62948 |

# Supplementary Figures


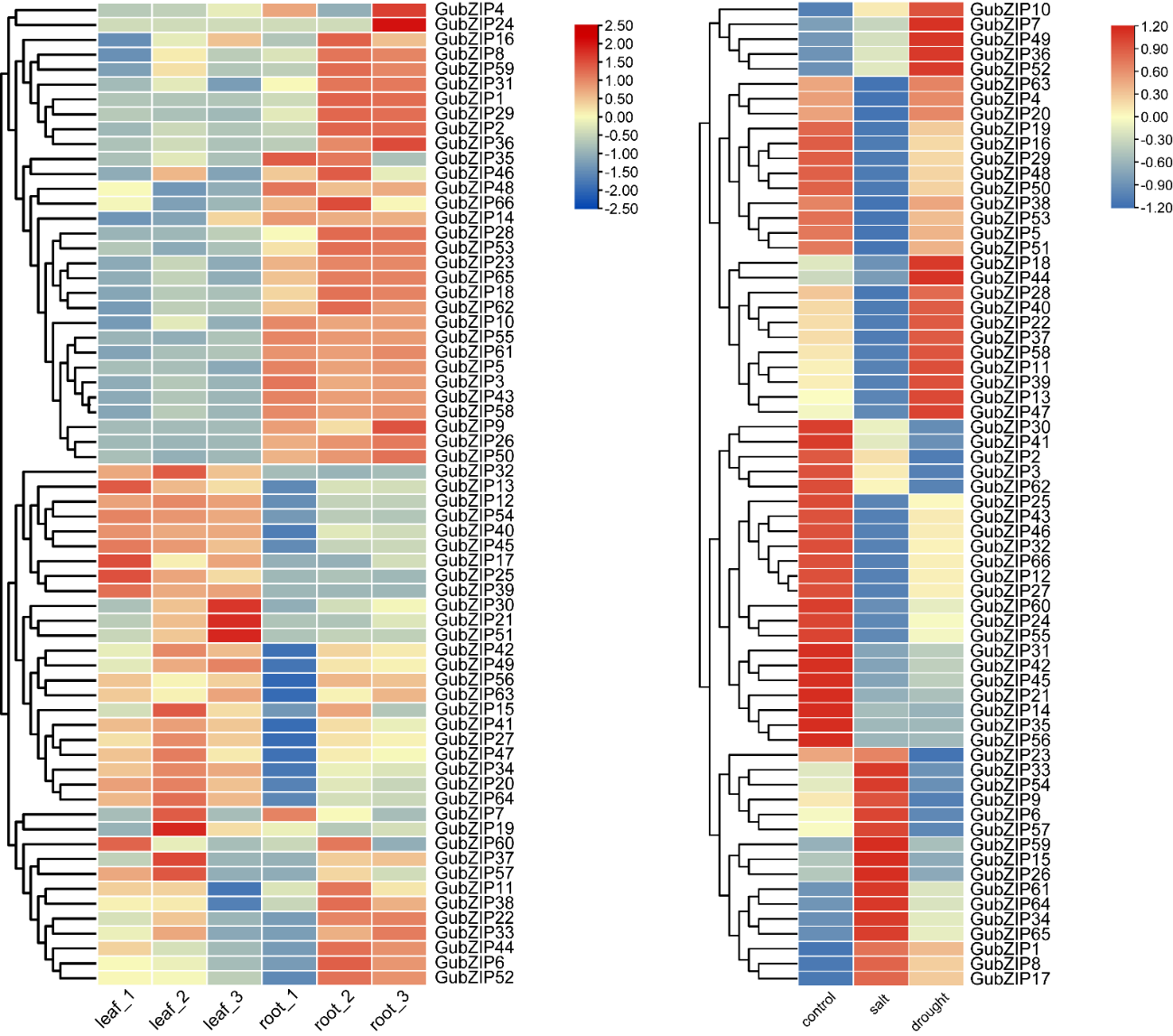


**Supplementary Figure 1 |** Spatial and abiotic stress expression patterns of *GubZIP* genes. Color scale indicates TPM normalized log2 conversion counts; blue indicates low expression and red indicates high expression. Expression data were obtained from the *Glycyrrhiza uralensis* genome database.
